# First-contact access from the perspective of users of primary health care: an evaluation in family and oral health teams

**DOI:** 10.31744/einstein_journal/2026AO2528

**Published:** 2026-08-14

**Authors:** Valmir Vanderlei Gomes, Lorrayne Belotti, Ana Carolina Cintra Nunes Mafra, Anne da Silva Almeida, Rebeca Cardoso Pedra, Fernanda Campos de Almeida Carrer

**Affiliations:** 1 Universidade de São Paulo Faculdade de Odontologia São Paulo SP Brazil Faculdade de Odontologia, Universidade de São Paulo, São Paulo, SP, Brazil.; 2 Hospital Israelita Albert Einstein Centro de Estudos, Pesquisa e Prática em Atenção Primária e Rede São Paulo SP Brazil Centro de Estudos, Pesquisa e Prática em Atenção Primária e Rede (CEPPAR), Hospital Israelita Albert Einstein, São Paulo, SP, Brazil.; 3 Hospital Israelita Albert Einstein Faculdade Israelita de Ciências da Saúde Albert Einstein São Paulo SP Brazil Faculdade Israelita de Ciências da Saúde Albert Einstein, Hospital Israelita Albert Einstein, São Paulo, SP, Brazil.; 4 Hospital Sírio-Libanês São Paulo SP Brazil Hospital Sírio-Libanês, São Paulo, SP, Brazil.

**Keywords:** Health services accessibility, Health services research, Oral health, Primary health care, Quality of health care

## Abstract

**Objective:**

In this study, we aimed to evaluate the primary health care attribute of first-contact access from the perspective of users of family and oral health teams in São Paulo, Brazil.

**Methods:**

A cross-sectional study was conducted at 12 basic health units between January and November 2021. The adult versions of the Primary Care Assessment Tool (PCATool-Brazil) and PCATool-Oral Health were used to assess first-contact access through its two components of utilization and accessibility.

**Results:**

In total, 537 individuals participated, including 129 users of oral health teams and 408 users of family health teams. In both groups, utilization scores were higher than accessibility scores. Among users of oral health teams, the mean scores were 8.08 for utilization and 4.37 for accessibility; among users of family health teams, the corresponding scores were 8.43 and 3.80. For oral and family health teams, high performance for utilization was observed in 85.37% and 87.53% of users, whereas low performance for accessibility was observed in 95.08% and 97.70% of users, respectively. Few differences in participant characteristics were observed among users of oral health teams, with higher utilization scores noted among those without private health insurance. Regarding users of family health teams, higher utilization scores were observed among older adults, retirees, or pensioners; beneficiaries of social programs; those without private health insurance; and those with hypertension and or diabetes. For accessibility, higher scores were observed among men, older adults, and retirees or pensioners.

**Conclusion:**

Overall, utilization was satisfactory and accessibility was unsatisfactory in both family and oral health teams. Primary health care services were recognized as a usual source of care, but organizational barriers to timely access remain, especially in oral health care. These findings highlight the need to strengthen accessibility in primary health care, particularly through organizational strategies aimed at improving timely access to care.

## Highlights

■Utilization scores were higher than accessibility scores in both family health teams and oral health teams.

■The performance of accessibility was predominantly low in both service modalities.

■Among oral health teams users, few differences were observed across participant characteristics.

■Utilization was higher among older, vulnerable, and chronically ill family health teams users.

## In Brief

Users of family and oral health teams recognized primary health care as a usual source of care, but accessibility remained a major barrier. Utilization was rated more positively than accessibility in both groups, with persistent difficulties experienced in organizing access to oral health care.

## Graphical abstract

**Figure:**
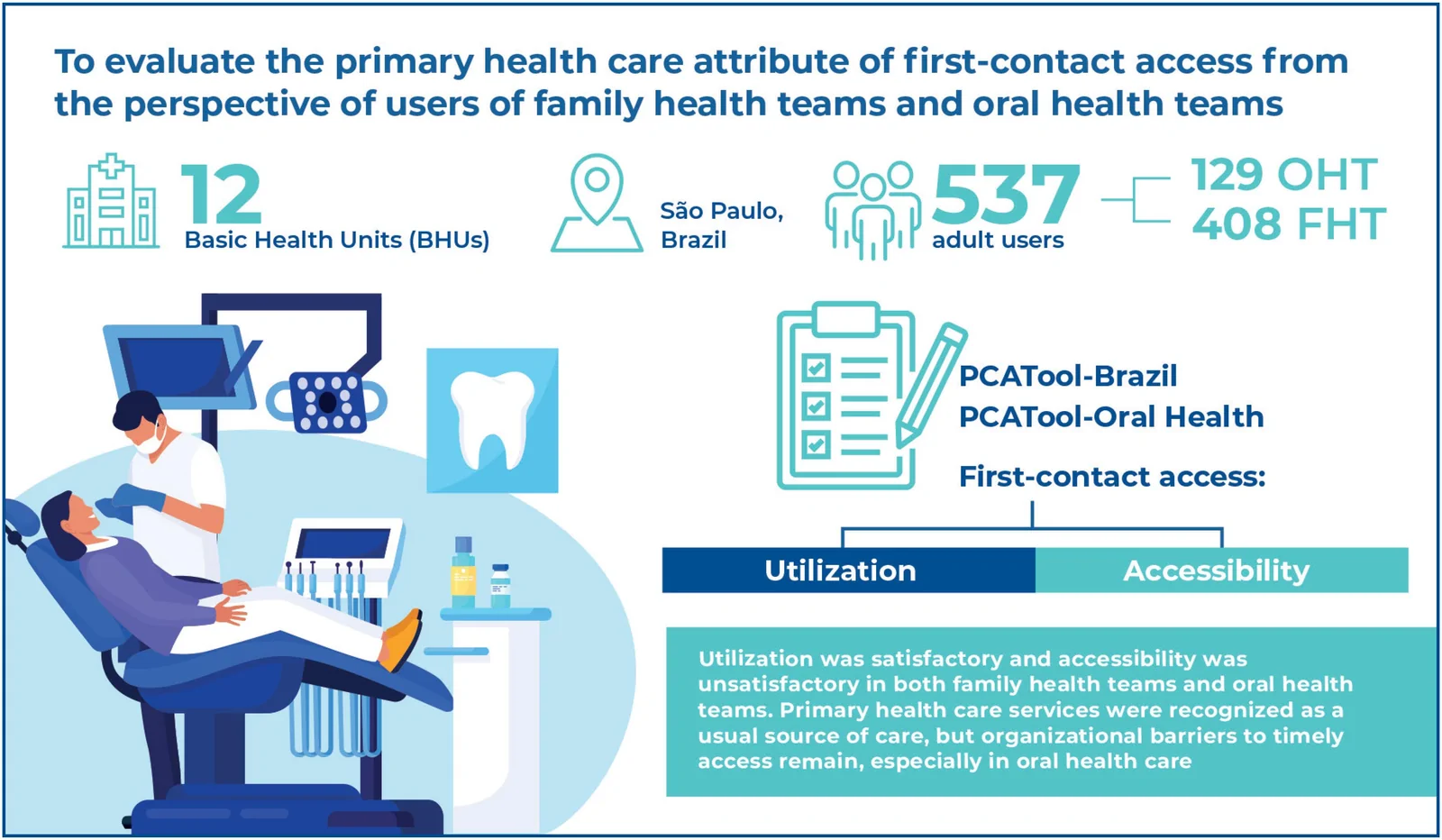

## INTRODUCTION

Primary health care (PHC) is structured around a set of essential attributes that guide the organization of health services and support the provision of comprehensive and continuous care.^(1,2)^ Among these attributes, first-contact access has a central role because it reflects whether PHC is recognized and used as the preferred entry point whenever a new health need arises or a new episode of an existing problem occurs.^(2)^ According to Starfield, this attribute comprises two complementary components: utilization, which refers to the use of the service as the usual source of care, and accessibility, which refers to the organizational conditions that enable users to obtain care when needed.^(2)^ If access is not adequately ensured, individuals may be prevented from benefiting from the other essential attributes of PHC.^(3)^

The relevance of strengthening PHC was reaffirmed in 2018 at the Global Conference on Primary Health Care held in Astana, Kazakhstan. At this conference, PHC was recognized as the most inclusive, effective, and efficient approach to improving population health and advancing universal health coverage.^(4)^ In this context, evaluating first-contact access is important for identifying barriers that limit timely entry into the health system and for supporting improvements in service organization.^(2,4)^

Among the instruments available for evaluating PHC, the Primary Care Assessment Tool (PCATool) is noteworthy because its use enables the core PHC attributes to be assessed from the users’ perspective and because it can be used in different evaluation contexts.^(5,6)^ In Brazil, the Ministry of Health published the manual for the PCATool-Brazil in 2020, consolidating the instrument as an important methodological reference for evaluating PHC in the Brazilian Public Health System (SUS - *Sistema Único de Saúde*).^(7)^ Although health outcomes cannot be directly assessed using the PCATool, it provides relevant information on the structure and process dimensions of care, supporting decision-making and the continuous improvement of services.^(6,7)^

In oral health, evaluating the attributes of PHC is particularly relevant because the incorporation of oral health teams (OHTs) into PHC has not always been accompanied by equivalent advances in the organization ofaccess and care.^(3)^ Evaluating first-contact access from the perspective of users of family health teams (FHTs) and OHTs may contribute to identifying gaps in how this PHC attribute is expressed across these two components of care.

## OBJECTIVE

To evaluate the primary health care attribute of first-contact access from the perspective of users of family and oral health teams in São Paulo, Brazil.

## METHODS

### Study design and setting

This was an observational, cross-sectional study conducted between January and November 2021 at 12 basic health units located in the southern region of the municipality of São Paulo, SP, Brazil, in the administrative districts of Campo Limpo and Vila Andrade. These districts comprise a socially vulnerable area with an estimated population of approximately 400,000 inhabitants, of whom approximately 301,000 were registered in the e-SUS system. In 2021, both districts were among the five areas with the highest proportion of households located in slums in the city of São Paulo (Vila Andrade, 32.7%; Campo Limpo, 21.7%).^(8)^

### Participants and data collection

Eligible participants were individuals aged 18 or older, of either sex, who had received care at their reference basic health unit. After completing the consultation or procedure in the health service, users were approached and invited to participate in the study. Those who agreed and provided informed consent completed the questionnaire. Users who did not have the physical or mental conditions required to answer the questionnaire, as well as those who declined to participate, were excluded. Participants were categorized according to the type of service used as users of OHTs or users of FHTs.

Data collection was carried out by professionals who were trained and calibrated prior to this study being conducted. The questionnaires were developed using the Research Electronic Data Capture (REDCap)^(9)^ platform and administered in electronic format to adult users of FHTs and OHTs. The sample size calculation was based on the analysis of all teams in a single analytical model, considering a significance level of 5% and statistical power of 90%. The total estimated sample was subsequently distributed among the teams included in the study.

### Instruments and variables

To assess PHC attributes in FHTs and OHTs, we used the adult user versions of the PCATool-Brazil and PCATool-Oral Health. In this study, the analysis focused on the PHC attribute of first-contact access, which reflects whether the service is recognized and used as the usual point of entry into care. In the PCATool, this attribute is operationalized through two components: utilization, related to the use of the service as the usual source of care, and accessibility, related to the organizational conditions that enable users to obtain care when needed. The structure of this attribute, including its components, corresponding item groups, and a brief description of their content, is presented in table 1. The same structure was adopted in both versions of the instrument.

**Table 1:** Items of the first-contact access attribute in the Primary Care Assessment Tool instruments

PHC attribute	PHC component	Items	Description of item content
First-contact access	Utilization	B1, B2, B3	Evaluates whether the basic health unit is recognized and used as the usual source of care and as the first service sought when a new health need arises or when routine follow-up is needed.
	Accessibility	C1, C2, C3, C4, C5, C6, C7, C8, C9, C10, C11, C12	Evaluates organizational aspects that may facilitate or hinder access to care, including appointment scheduling, waiting time, service availability, opening hours, and the possibility of receiving care or guidance when needed.

Source: Brasil. Ministério da Saúde. Departamento de Atenção Primária à Saúde. Manual da Ferramenta de Avaliação da Atenção Primária (PCATool-Brasil). Brasília (DF): Ministério da Saúde; 2020 [citado 2023 Out 26]. Disponível em: https://aps.saude.gov.br/biblioteca/visualizar/MjAwMg==

PHC: primary health care.

In addition to the PCATool instruments, a semi-structured questionnaire was administered to collect sociodemographic and clinical information, including sex, age, self-reported race/ethnicity, educational level, employment status, receipt of government social benefits, private health insurance coverage, and self-reported history of hypertension and diabetes.

### Score construction

Responses were recorded on a Likert-type scale ranging from 1 to 4: 1 (*definitely no*), 2 (*probably no*), 3 (*probably yes*), and 4 (*definitely yes*), as well as 9 (*do not know/do not remember*). After data processing, scores for the first-contact access components of utilization and accessibility were calculated according to the recommendations of the PCATool manual and converted to a 0 to 10 scale. Scores of ≥6.6 were interpreted as high, indicating an adequate extent of the attribute, whereas scores of <6.6 were interpreted as low.^(7)^

### Statistical analysis

For the statistical analysis, the dependent variables were the scores of the two components of the first-contact access attributes, namely, utilization and accessibility, analyzed separately for OHT and FHT users. The independent variables were the users’ sociodemographic and clinical characteristics. Categorical variables were presented as absolute and relative frequencies, and continuous variables were reported as means and standard deviations. Normality of score distributions was assessed using the Shapiro-Wilk test to guide the selection of the most appropriate statistical tests. Variables with normal distribution were analyzed using Student's *t* test for comparisons between two groups and one way analysis of variance for comparisons between three or more groups. Variables without normal distribution were analyzed using the Mann-Whitney U and Kruskal-Wallis tests. The analyses were performed using RStudio and a p<0.05 was considered statistically significant. This procedure enabled the analytical strategy to be defined according to the distributional properties of each variable.

### Ethical aspects

This study was approved by the Research Ethics Committees of *Hospital Israelita Albert Einstein* (CAAE: 06807019.2.0000.0071; #4,034,263), the *Secretaria Municipal de Saúde de São Paulo*, SP (CAAE: 06807019.2.3001.0086; #3,263,871), and the *Faculdade de Ondontologia* of the *Universidade de São Paulo* (CAAE: 69599423.3.0000.0075; # 6,104,037).

## RESULTS

A total of 537 individuals participated in the study, including 129 users of OHT and 408 users of FHT. Among OHT users, female participants (71.32%) and individuals younger than 45 years (66.96%) predominated. Most participants self-identified as brown (44.53%), had completed education up to the level of high school (50.39%), were unemployed (40.31%), did not receive government social benefits (78.91%), and did not have private health insurance (82.81%). Most also reported no difficulties in seeing, hearing, or walking (50.78%), had no reported diseases (60.47%), and did not report hypertension or diabetes (73.64%; Table 2).

**Table 2:** Descriptive characteristics of those study participants who evaluated oral (n=129) and family (n=408) health teams

Variable		OHT Group n (%)	FHT Group n (%)
Sex			
	Male	37 (28.68)	73 (17.89)
	Female	92 (71.32)	335 (82.11)
Age (years)			
	Younger than 45 years	75 (66.96)	227 (56.05)
	45 years and older	37 (33.04)	178 (43.95)
Self-declared race/ethnicity			
	White	43 (33.59)	135 (33.17)
	Black	23 (17.97)	71 (17.44)
	Brown	57 (44.53)	189 (46.44)
	Other/do not know/prefer not to say	5 (3.91)	12 (2.95)
Education level			
	Up to middle school	45 (34.88)	156 (38.42)
	Up to high school	65 (50.39)	184 (45.32)
	College degree and postgraduate	19 (14.73)	66 (16.26)
Employment status			
	Unemployed	52 (40.31)	147 (36.30)
	Self-employed	24 (18.60)	63 (15.56)
	Salaried with formal employment	35 (27.13)	111 (27.41)
	Salaried without formal employment	9 (6.98)	28 (6.91)
	Retired/pensioner	9 (6.98)	56 (13.83)
Government social benefits			
	No	101 (78.91)	249 (62.56)
	Yes	27 (21.09)	149 (37.44)
Private health insurance				
	No	106 (82.81)	364 (91.23)
	Yes	22 (17.19)	35 (8.77)
Reported difficulties in seeing, hearing, or walking				
	No	65 (50.78)	159 (40.05)
	Yes	63 (49.22)	238 (59.95)
Number of reported diseases				
	None	78 (60.47)	151 (37.01)
	One or two	37 (28.68)	175 (42.89)
	More than two	14 (10.85)	82 (20.10)
Hypertension/diabetes			
	No	95 (73.64)	254 (62.25)
	Yes	34 (26.36)	154 (37.75)

FHT: family health team; OHT: oral health team.

Among FHT users, female participants also predominated (82.11%), as did individuals younger than 45 years (56.05%). Most participants self-identified as brown (46.44%), had completed education up to the level of high school (45.32%), were unemployed (36.30%), did not receive government social benefits (62.56%), and did not have private health insurance (91.23%). In this group, most participants reported difficulties in seeing, hearing, or walking (59.95%), had one or two reported diseases (42.89%), and did not report hypertension or diabetes (62.25%; Table 2).

The mean scores and performance of the first-contact access components according to service type are presented in table 3. In both groups, higher mean scores were achieved for utilization than for accessibility. Among OHT users, the mean scores were 8.08 and 4.37 for utilization and accessibility, respectively. Among FHT users, the mean scores were 8.43 and 3.80, respectively. A high performance in utilization was observed in 85.37% of OHT users and in 87.53% of FHT users, whereas a low performance in accessibility was observed in 95.08% and 97.70% of users, respectively.

**Table 3:** Scores and performance of the first-contact access attribute according to utilization and accessibility* components

		Mean (SD)	Minimum-maximum	High performance n (%)	Low performance n (%)
Oral health, adults					
	First contact access score-utilization	8.08 (2.37)*	0.00-10.00	105 (85.37)	18 (14.63)
	First contact access score-accessibility	4.37 (1.25)	1.11-7.22	6 (4.92)	116 (95.08)
Family health, adults					
	First contact access score-utilization	8.43 (2.29)*	0.00-10.00	351 (87.53)	50 (12.47)
	First contact access score-accessibility	3.80 (1.67)	0.00-8.06	9 (2.30)	382 (97.70)

* A high score was defined as a score of ≥6.6.

The mean utilization and accessibility scores among OHT users according to the independent variables are shown in table 4. Among OHT users, individuals without private health insurance had significantly higher utilization scores than those with private health insurance (8.33 *versus* 6.83; p=0.019). No statistically significant differences were observed for accessibility.

**Table 4:** Mean utilization and accessibility scores among users of oral health teams according to independent variables

Variable		Utilization		Accessibility	
		Mean (SD)	p value	Mean (SD)	p value
Sex					
	Male	8.18 (2.88)	0.238*	4.36 (1.05)	0.960**
	Female	8.04 (2.17)		4.38 (1.32)	
Age (years)					
	Younger than 45 years	7.76 (2.58)	0.053*	4.35 (1.33)	0.538**
	45 years and older	8.76 (1.59)		4.20 (1.17)	
Self-declared race/ethnicity					
	White	7.65 (2.82)	0.089†	4.37 (0.97)	0.335††
	Black	7.37 (2.71)		4.78 (1.08)	
	Brown	8.66 (1.69)		4.25 (1.44)	
	Other/do not know/prefer not to say	9.17 (1.06)		3.89 (1.67)	
Education level					
	Up to middle school	8.62 (1.47)	0.362†	4.34 (1.39)	0.571††
	Up to high school	7.92 (2.64)		4.33 (1.13)	
	College degree and postgraduate	7.34 (2.85)		4.60 (1.34)	
Employment status					
	Unemployed	8.50 (2.05)	0.381†	4.57 (1.28)	0.294††
	Self-employed	8.24 (2.22)		4.07 (1.28)	
	Salaried with formal employment	7.36 (2.82)		4.40 (1.31)	
	Salaried without formal employment	7.78 (3.09)		3.86 (0.85)	
	Retired/pensioner	8.15 (1.57)		4.68 (0.76)	
Government social benefits					
	No	8.09 (2.40)	0.815*	4.36 (1.25)	0.862**
	Yes	7.98 (2.35)		4.41 (1.28)	
Private health insurance					
	No	8.33 (2.15)	0.019*	4.38 (1.28)	0.947**
	Yes	6.83 (3.01)		4.40 (1.11)	
Reported difficulties in seeing, hearing, or walking					
	No	8.05 (2.61)	0.688†	4.45 (1.27)	0.455**
	Yes	8.12 (2.12)		4.28 (1.22)	
Number of reported diseases					
	None	7.90 (1.72)	0.082†	3.93 (0.94)	0.439††
	One or two	8.09 (2.48)		4.41 (1.27)	
	More than two	9.17 (2.26)		4.44 (1.30)	
Hypertension/diabetes					
	No	7.85 (2.51)	0.063*	4.39 (1.29)	0.778**
	Yes	8.75 (1.74)		4.32 (1.11)	

* Mann-Whitney U test

† Kruskal-Wallis test

** Student's t-test

†† Analysis of variance test.

The corresponding analyses for FHT users are shown in table 5. In this group, higher utilization scores were observed among individuals aged 45 years or older (8.70; p=0.003), retirees or pensioners (9.09; p=0.001), beneficiaries of government social programs (8.62; p=0.047), those without private health insurance (8.53; p=0.036), and those with hypertension and or diabetes (8.86; p=0.001). Significantly higher mean scores were observed for accessibility among male participants (4.15; p=0.047), individuals aged 45 years or older (3.93; p=0.048), and retirees or pensioners (4.39; p<0.001).

**Table 5:** Mean utilization and accessibility scores among users of family health teams according to independent variables

Variable		Utilization		Accessibility	
		Mean (SD)	p value	Mean (SD)	p value
Sex					
	Male	8.58 (2.51)	0.199*	4.15 (1.72)	0.047*
	Female	8.40 (2.24)		3.73 (1.66)	
Age (years)					
	Younger than 45 years	8.22 (2.29)	0.003*	3.68 (1.65)	0.048*
	45 years and older	8.70 (2.29)		3.93 (1.70)	
Self-declared race/ethnicity					
	White	8.60 (2.36)	0.095†	4.00 (1.56)	0.346†
	Black	8.34 (2.16)		3.53 (2.00)	
	Brown	8.41 (2.29)		3.78 (1.60)	
	Other/do not know/prefer not to say	7.41 (2.29)		3.42 (1.98)	
Education level					
	Up to middle school	8.69 (2.05)	0.099*	3.76 (1.64)	0.843*
	Up to high school	8.45 (2.23)		3.81 (1.64)	
	College degree and postgraduate	7.78 (2.84)		3.88 (1.87)	
Employment status					
	Unemployed	8.37 (2.39)	0.001†	3.74 (1.68)	<0.001†
	Self-employed	8.89 (1.87)		4.28 (1.44)	
	Salaried with formal employment	7.93 (2.46)		3.49 (1.73)	
	Salaried without formal employment	8.29 (1.89)		2.85 (1.55)	
	Retired/pensioner	9.09 (2.11)		4.39 (1.55)	
Government social benefits					
	No	8.30 (2.32)	0.047*	3.81 (1.69)	0.798*
	Yes	8.62 (2.28)		3.77 (1.67)	
Private health insurance					
	No	8.53 (2.21)	0.036*	3.80 (1.66)	0.999*
	Yes	7.46 (2.92)		3.77 (1.80)	
Reported difficulties in seeing, hearing, or walking					
	No	8.20 (2.49)	0.203*	3.88 (1.64)	0.609*
	Yes	8.56 (2.17)		3.77 (1.69)	
Number of reported diseases					
	None	8.83 (1.96)	0.094†	3.67 (1.82)	0.763†
	One or two	8.06 (2.68)		3.82 (1.61)	
	More than two	8.57 (2.02)		3.85 (1.66)	
Hypertension/diabetes					
	No	8.18 (2.41)	0.001*	3.71 (1.65)	0.061*
	Yes	8.86 (2.02)		3.96 (1.72)	

* Mann-Whitney U test

† Kruskal-Wallis test.

## DISCUSSION

The findings of this study showed that, in both family and oral health teams, the utilization component of first-contact access was more positively evaluated than the accessibility component. Overall, this suggests that PHC services were recognized by users as a usual source of care, but important organizational barriers still limited timely access. The results also indicated that a more consistent pattern of differences in sociodemographic and clinical characteristics was present among FHT users than among OHT users. Together, these findings highlight that, although PHC services appear to be an established point of entry into care, accessibility is a persistent challenge, particularly in oral health care.

Similar results have been reported in previous studies conducted in Brazilian PHC settings, especially with regard to difficulties related to service hours, appointment scheduling, and time spent waiting for care.^(3,10,11)^ Recent Brazilian studies have also shown that, from the user's perspective, first-contact access—particularly for the accessibility component—tends to be among the most fragile of the PHC attributes, even in services otherwise oriented toward primary care.^(12,13)^ This convergence reinforces the interpretation that the main challenges lie in recognizing PHC as a usual source of care, as well as in ensuring timely and feasible access under everyday service conditions.

First-contact access is a core PHC attribute because it conditions the user's entry into the health system and influences the possibility of benefiting from the other essential attributes of care.^(2,3)^ In this sense, the more favorable performance observed for utilization may reflect the role of PHC services as a regular point of care, particularly for follow-up and routine health needs. By contrast, the low scores observed for accessibility in both groups indicate persistent barriers in the organization of care, reinforcing the need to improve service availability and responsiveness to users’ needs.^(7,14)^

Among OHT users, few statistically significant differences were observed in participants’ sociodemographic and clinical characteristics. The only significant association was found for private health insurance coverage, with higher utilization scores among users without private health insurance. This finding may reflect a greater dependence on public oral health services among individuals who rely exclusively on the SUS. It also suggests that this population is using oral health care as a reference source of care, although this did not translate into better accessibility scores. The limited variation observed across OHT subgroups should not be interpreted as evidence of more equitable access; rather, it may reflect a broadly low level of accessibility across different user profiles.

Among FHT users, more positive evaluations of utilization were observed among older adults, retirees or pensioners, beneficiaries of social programs, those without private health insurance, and those with hypertension and or diabetes. This finding suggests that PHC has been functioning more consistently for patients with greater health needs and for those who depend more directly on public services. The result is compatible with the programmatic role of PHC in the longitudinal management of chronic conditions, which requires more regular contact with the health service and may strengthen its recognition as the usual source of care.^(15,16)^ However, better evaluation of utilization among these groups should not be interpreted as the absence of barriers, but rather as evidence of stronger insertion into established care flows and follow-up routines.

Regarding accessibility among FHT users, more positive evaluations were observed among men, older adults, and retirees or pensioners. These findings may be related, at least in part, to greater compatibility between the opening hours of basic health units and the daily routines of these groups. However, the low scores observed overall for accessibility indicate that this component remains fragile, even among subgroups with relatively more favorable evaluations. Expanding service hours and adopting strategies that facilitate adults of working age gaining access may help reduce these barriers.^(17)^ Similar concerns have been raised in recent Brazilian studies, in which persistent weaknesses in first-contact access despite broader PHC orientation have been noted.^(12,13)^

The findings in oral health deserve particular attention. Although OHT users reported satisfactory utilization scores, accessibility remained low, and few subgroup differences were identified. This may indicate that, in oral health care, difficulties continue to be faced in organizing access in accordance with a broader PHC approach and in the context of the growing emphasis on oral health within universal health coverage.^(18,19)^ In practice, oral health services may still be more strongly oriented toward responding to immediate or procedure-centered demands than toward the comprehensive and timely organization of care within PHC.^(20,21)^ Furthermore, the weaker differentiation observed among user subgroups may suggest that barriers to accessibility are distributed more uniformly, rather than concentrated in specific profiles.

This study had some limitations that should be considered when interpreting the results. First, its cross-sectional design does not allow causal inferences. Second, the PCATool assesses the structure and process dimensions of care, but does not directly evaluate health outcomes.^(14)^ The study may also have been subject to selection bias, because the sample included only individuals who were able to access the basic health unit and complete care. Consequently, the experiences of users who may have faced more severe barriers—such as being unable to schedule an appointment, failing to obtain care, or dropping out before receiving care—were not captured. This may have led to an overestimation of first-contact access, particularly for the utilization component.

Despite these limitations, the study provides relevant evidence on first-contact access from the perspective of PHC users in family and oral health teams. These findings may support service planning and management, especially in efforts aimed at reducing organizational barriers and strengthening access as a core PHC attribute.

## CONCLUSION

The evaluation conducted using the Primary Care Assessment Tool showed that, in both family and oral health teams, the utilization component of first-contact access was more positively assessed by users than the accessibility component. These findings suggest that primary health care services are recognized by users as a usual source of care, but that they still face important organizational barriers to timely access.

Among family health teams users, the findings indicated more positive evaluations of utilization among groups with greater health needs and greater dependence on public services. Conversely, among oral health teams users, fewer differences in user characteristics were observed, and accessibility remained low. Overall, the findings suggest persistent challenges in organizing access in oral health care in accordance with a broader primary health care approach. These findings reinforce the importance of strengthening accessibility in primary health care, particularly through organizational strategies that facilitate timely access and reduce barriers related to service availability and functioning.

## Data Availability

Data cannot be made publicly available. Enter a justification: The dataset underlying this manuscript contains sensitive human participant information derived from health records and/or individual-level research data. Due to ethical, legal, and confidentiality restrictions, these data cannot be made publicly available. Public sharing could compromise participant privacy, even after de-identification, given the nature and context of the information. Data may be made available only upon reasonable request, subject to approval by the responsible ethics committee/institution and in accordance with applicable data protection and confidentiality regulations.
